# *Heteropolygonatum xishuiense* (Asparagaceae, Convallarioideae, Polygonateae), a new species from Danxia cliffs in Guizhou, China

**DOI:** 10.3897/phytokeys.271.182884

**Published:** 2026-02-10

**Authors:** Xiao Wang, Miao-Miao Wang, Mao-Qin Xia, Qin Tian, Chong-Qing Li, Jun Mu, Meng-Qi Han

**Affiliations:** 1 Guizhou Xishui National Nature Reserve Management Bureau, Xishui, Guizhou, China College of Smart Agriculture, Chongqing University of Arts and Sciences Chongqing China; 2 China National Botanical Garden (North Garden), Beijing, China Kunming Institute of Botany, Chinese Academy of Sciences Kunming China; 3 Chongqing Key Laboratory for Germplasm Innovation of Special Aromatic Spice Plants, College of Smart Agriculture, Chongqing University of Arts and Sciences, Chongqing, China China National Botanical Garden (North Garden) Beijing China; 4 Honghe Tropical Agriculture Institute of Yunnan, Hekou, Yunnan, China Guizhou Xishui National Nature Reserve Management Bureau Xishui China; 5 State Key Laboratory of Plant Diversity and Specialty Crops, Kunming Institute of Botany, Chinese Academy of Sciences, Kunming, Yunnan, China Honghe Tropical Agriculture Institute of Yunnan Hekou China

**Keywords:** Danxia landform, Polygonateae, taxonomy

## Abstract

*Heteropolygonatum xishuiense* (Asparagaceae, Convallarioideae, Polygonateae), a new microendemic species from southwest China, is described and illustrated based on morphological comparisons and molecular phylogenetic analyses. Phylogenetic analyses based on the chloroplast genome indicate that this new species is sister to *H. ogisui*. Morphologically, *H. xishuiense* is similar to *H. ginfushanicum* and *H. ogisui*.

## Introduction

In a systematic taxonomic study of the genus *Polygonatum*Miller (1754), Tamura and Ogisu established the genus *Heteropolygonatum* M.N.Tamura & Ogisu (1997) on the basis of several characteristics, such as short outer stamens and long inner stamens, imbricate perianth segments, and terminal and axillary inflorescences (Tamura et al. 1997). Karyological studies have also revealed that *Heteropolygonatum* exhibits a bimodal karyotype characterized by a basic chromosome number of x = 16, which differs from *Polygonatum* (Tamura et al. 1997; Yamashita and Tamura 2001; Tamura et al. 2008; Floden 2014a; Floden 2014b; Floden and Pendry 2025; Masuda et al. 2025). This classification was further supported by molecular evidence confirming *Heteropolygonatum* and *Polygonatum* as monophyletic sister groups (Floden and Schilling 2018; Gu et al. 2021; Wang et al. 2022; Xia et al. 2022; Zhang et al. 2023; Hu et al. 2025). Based on these studies, several species have been transferred to *Heteropolygonatum* (Tamura et al. 2000; Chao et al. 2013; Floden 2014b; Zhu et al. 2022; Zhou et al. 2025), while a species of *Polygonatum* erroneously described in *Heteropolygonatum* was removed as a correction (Floden 2014a). In addition, new taxa have been described (Tamura and Xu 2004; Xiao et al. 2017; Floden 2018; Floden and Pendry 2025). Following these taxonomic studies, *Heteropolygonatum* currently comprises 14 species. Most are distributed in China, while some have also been discovered in mountainous regions of Vietnam and Myanmar adjacent to China (Floden 2014b; Floden and Pendry 2025). Most species in this genus have extremely limited distributions, often found only at a single location or on a few peaks within a mountain range.

During ongoing long-term biodiversity surveys in the Xishui Nature Reserve, which is characterized primarily by the Danxia landform, an unknown plant of the tribe Polygonateae was occasionally observed on some cliffs. After several years of tracking, it was observed flowering for the first time in May 2025. Its imbricate petals and terminal inflorescence suggested that it might belong to *Heteropolygonatum*. Through specimen examination and literature review, this taxon could not be identified as any known species of *Heteropolygonatum*. Supported by evidence from molecular phylogenetic studies based on chloroplast genome data, we are confident that it represents a previously undescribed new species. Here, we name it *Heteropolygonatum xishuiense* and provide its description and illustration.

## Material and method

In May 2025, living specimens of the plant in its flowering period were first collected in the Guizhou Xishui National Nature Reserve. Field-fresh materials were dissected on-site and photographed, while parts of the plants were pressed into herbarium specimens, which are deposited at KUN. We examined herbarium specimens of conspecific genera from herbaria (CDBI, GACP, GZAC, GZTM, IBSC, KUN, PE) and reviewed images of additional specimens through the Global Biodiversity Information Facility (https://www.gbif.org/) and the Chinese Virtual Herbarium (https://www.cvh.ac.cn/). Simultaneously, comparisons were made with published taxonomic literature on species within the same genus.

In this study, a new plastid sequence, the complete chloroplast genome of *Heteropolygonatum xishuiense*, was generated. Genomic DNA was extracted from silica gel-dried leaves using a modified CTAB method (Doyle and Doyle 1987). The genomic DNA was sequenced on the BGISEQ-500 platform (BGI, China) following the manufacturer’s standard protocols. Briefly, libraries were prepared using the Hieff NGS^®^ OnePot Pro DNA Library Prep Kit V4 (Cat. No. 12972). Sequencing was performed with 150 bp paired-end reads to generate raw data. Approximately 12 Gb of raw data was generated for each sample from genome skimming. All raw data were trimmed by removing adapters and low-quality reads and then used for assembling plastomes. Quality control and data processing were conducted using Fastp v0.24.0 (Chen 2023). The plastid genome was assembled de novo using GetOrganelle v1.7.7.1 (Jin et al. 2020).

All 20 plastid sequences of *Heteropolygonatum* were downloaded from NCBI, along with *Polygonatum hirtellum* (PQ436973) and *Disporopsis jinfushanensis* (MH891733) as outgroups (Fig. 2). The two assembled sequences were initially aligned with the sequences downloaded from NCBI using MAFFT v7.450 (Katoh et al. 2002; Katoh and Standley 2013) in Geneious v10.2.6. The alignment result that matched the Large Single Copy (LSC) and Small Single Copy (SSC) directions of the NCBI sequences was selected and realigned. Manual inspection of the alignment revealed no significant structural variations among the plastid genomes of these taxa. Therefore, the alignment results were manually trimmed in Geneious v10.2.6 to ensure a consistent starting region. The sequences were realigned using MAFFT v7.450, and the alignment result was used to construct a maximum likelihood tree in IQ-TREE v2.4.0 with the parameters “--alrt 1000 -B 1000” (Nguyen et al. 2014). The resulting maximum likelihood tree was visualized using FigTree v1.4.1 (available at: http://tree.bio.ed.ac.uk/software/figtree/).

## Taxonomic treatment

### Heteropolygonatum xishuiense

Taxon classificationPlantaeAsparagalesAsparagaceae

Xiao Wang bis & M.Q.Han sp. nov.

EE97DDF1-620C-5567-A4BE-BE229361F018

urn:lsid:ipni.org:names:77376566-1

Fig. 1

#### Diagnosis.

*Heteropolygonatum xishuiense* is related to *H. ginfushanicum* (FT Wang & T. Tang) M.N.Tamura, S.C.Chen & Turland (Tamura et al. 2000) and *H. ogisui* M.N.Tamura & J.M.Xu (2004), but differs by its cylindrical rhizomes (vs. moniliform in *H. ogisui*), stems green, densely purple maculate (vs. stems reddish to purple in *H. ginfushanicum*), leaves 2–6, succulent, abaxially purple, with veins other than the midvein inconspicuous (vs. leaves 2, abaxially green, with 7 veins in *H. ginfushanicum*; leaves 2–5, papery, abaxially dull white, with 7 veins in *H. ogisui*), inflorescence terminal and axillary (vs. inflorescence only terminal at the terminal leaf in *H. ginfushanicum*), and flowers white (vs. flowers white pale green in *H. ginfushanicum*; flowers purple in *H. ogisui*) (Fig. 3; Table 1).

**Figure 1.**
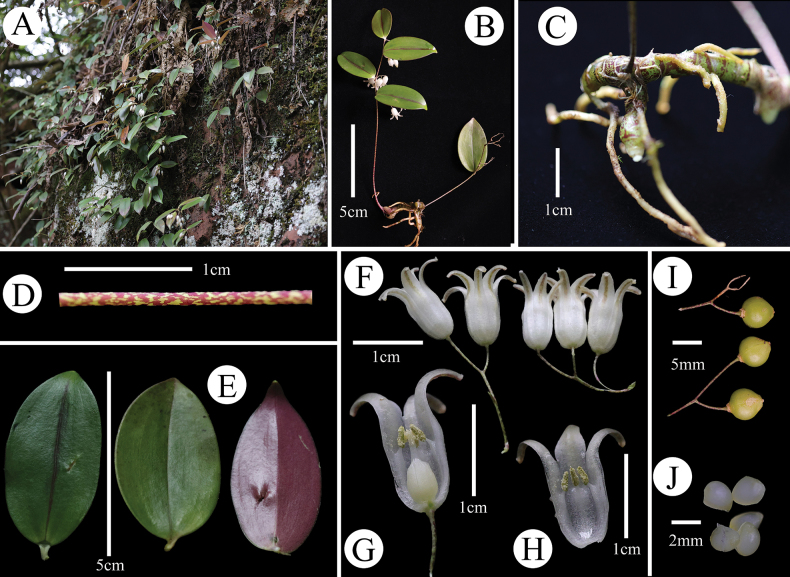
*Heteropolygonatum xishuiense* sp. nov. **A**. Habitat; **B**. Habit; **C**. Rhizome; **D**. Stem; **E**. Leaf adaxial surface and leaf abaxial surface; **F**. Flowers; **G, H**. Longitudinally cut corolla; **I**. Berries; **J**. Seeds.

**Figure 2.**
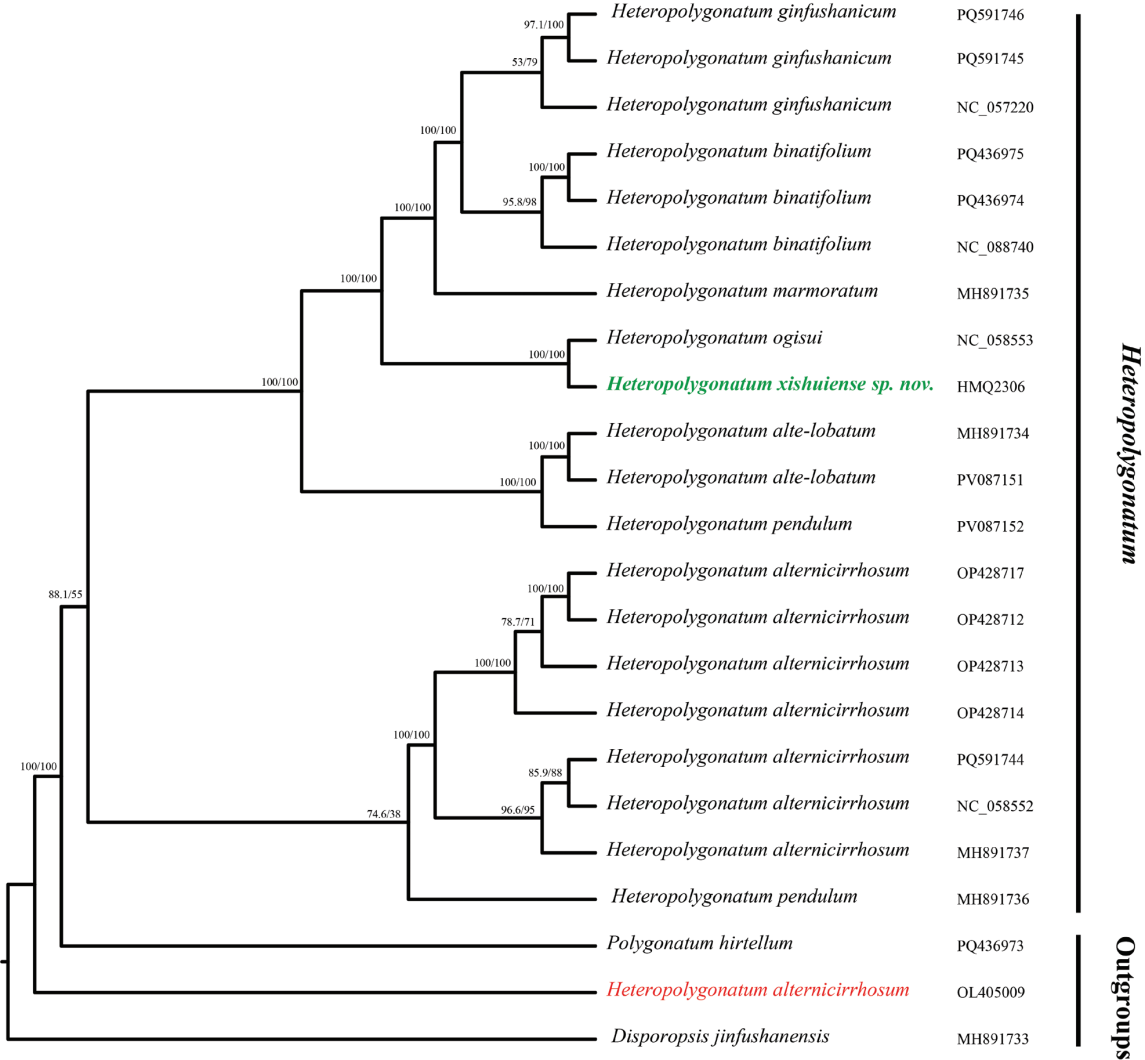
Maximum likelihood tree constructed based on whole chloroplast genome sequences. *Heteropolygonatum xishuiense* is highlighted in green and bold. The numbers above and below the branches represent support values from the SH-aLRT test and ultrafast bootstrap, respectively. The data for OL405009 (red) were published in Wang et al. (2022), but there is a potential misidentification issue with this sample.

**Figure 3.**
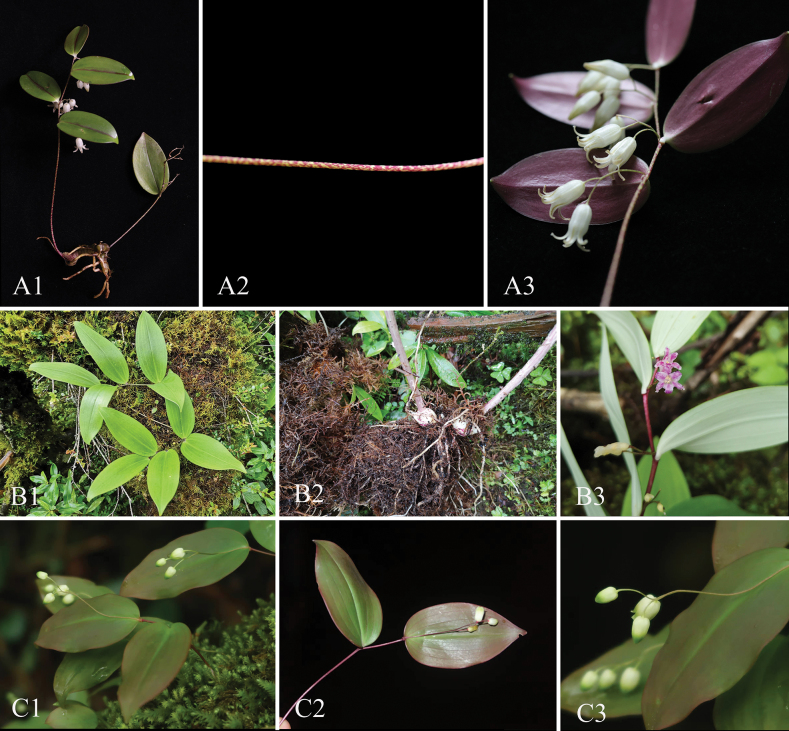
*Heteropolygonatum xishuiense* (**A1–A3**), *H. ogisui* (**B1–B3**), and *H. ginfushanicum* (**C1–C3**).

**Table 1.** Comparison of morphological characteristics of *Heteropolygonatum xishuiense*, *H. ogisui*, and *H. ginfushanicum*.

Character	*H. xishuiense*	*H. ogisui*	*H. ginfushanicum*
Plant height	10–20 cm	10–22 cm	5–14 cm
Rhizomes	Cylindrical	Moniliform	Cylindrical
Stems	many, clustered; green, densely covered with purple markings	single to few; pale green, with purple speckled	many, clustered; purple
Leaves	2–6; adaxially green, partly purple along midrib, abaxially purple; leaf base cuneate	2–5; adaxially pale green, abaxially dull white; leaf base cuneate	2; adaxially pale purple, abaxially green; leaf base rounded
Flowers	2–4 flowers; white	1–2 flowers; purple	2–4 flowers; pale green

#### Type.

China • Guizhou Province, Zunyi City, Xishui County, Xishui National Nature Reserve, 28°09'N, 105°54'E, 1200 m a.s.l., 21 April 2024, *Meng-Qi Han, Xiao Wang, Miao-Miao Wang & Qi An HMQ2306* (holotype: KUN [1729525]!, isotypes: PE!, IBK!).

#### Description.

Perennial lithophytic ***herb***, up to 20 cm tall. ***Rhizome*** tuberous, purple-mottled, 5–10 mm in diameter, with numerous fibrous roots along its length. ***Stem*** erect, unbranched, densely purple-maculate, glabrous, ca. 1 mm in diameter. ***Leaves*** 2–6, alternate; ***blade*** succulent, ovate, 3–5 cm long, 2–4 cm wide, except for midvein, other veins inconspicuous, adaxially green, abaxially purple, both sides glabrous, apex acuminate, base broadly cuneate and decurrent into petiole; petiole 2–5 mm long; midvein adaxially impressed, abaxially raised. ***Inflorescence*** terminal and axillary, 2–4-flowered; pedicels 2–3 cm long, terete; peduncle 3–10 mm long; peduncle and pedicels glabrous, densely purple-mottled; bracts absent. ***Flowers*** white, 10–15 mm long, corolla tube 4–5 mm in diameter, limb 5–12 mm in diameter; tube 5–8 mm long; ***tepals*** 6, linear-oblong, arranged in two whorls of three, 2–3 mm wide, glabrous, apex acute to obtuse, imbricate, fused for half or slightly more, lobes 5–7 mm long, white, abaxially light green along the midrib of the lobes, recurved. ***Stamens*** 6, adnate to perianth lobes, arranged in two whorls, inner whorl ca. 3 mm long, outer whorl ca. 2.5 mm long, free filament part ca. 2 mm long, smooth; anthers lanceolate, ca. 2 mm long, ca. 0.8 mm wide, apex acute, 2-loculed, longitudinally dehiscent, introrse. ***Ovary*** superior, ovoid, ca. 5 mm long, ca. 3 mm in diameter, glabrous; style slender, ca. 4 mm long; stigma glabrous. ***Berries*** globose, glabrous, 3-loculed, 5–8 mm in diameter. ***Seeds*** 6, ovoid, slightly flattened, 2–2.2 mm long, 1.8–2 mm wide.

#### Phenology.

This species was observed flowering from April and fruiting in July.

#### Etymology.

The specific epithet is derived from the type locality, Xishui County, Zunyi City, Guizhou Province, China.

#### Vernacular name.

Chinese Mandarin: xí shuǐ yì huáng jīng (习水异黄精).

#### Distribution, habitat, and ecology.

*Heteropolygonatum xishuiense* is currently known only from the Danxia landform cliffs within the Guizhou Xishui National Nature Reserve, where it occurs along ridges in montane broad-leaved forests at elevations of approximately 1,000–1,400 m. The species is restricted to north-facing, shaded sandstone cliffs characteristic of the Danxia landform. Individuals grow on nearly vertical rock surfaces that are extensively covered by mosses, which likely facilitate moisture retention and substrate stability. Owing to frequent precipitation and high humidity, these moss-covered rock faces remain consistently moist during the rainy season, creating a cool and humid microhabitat.

#### Conservation status.

*Heteropolygonatum xishuiense* is a rare species with a highly restricted geographical range and a limited population size. It is currently known only from within the Guizhou Xishui National Nature Reserve, where it grows on Danxia landform cliffs composed of reddish sandstone and conglomerate rocks. This habitat shares similarities with limestone karst features where other *Heteropolygonatum* species are known. Based on the IUCN Red List Categories and Criteria (Version 16) (IUCN 2024), the species meets Criterion D (very small or restricted population), with the total number of mature individuals estimated to be fewer than 250 across its three known subpopulations. Therefore, we provide a preliminary assessment of Endangered (EN).

#### Discussion.

The maximum likelihood tree reconstructed based on whole chloroplast genome sequences strongly supports *Heteropolygonatum xishuiense* and *H. ogisui* as sister species. Morphologically, *H. xishuiense* is more similar to *H. ginfushanicum*, and its distributional range is also geographically closer to that of *H. ginfushanicum*.

Additionally, the data from Wang et al. (2022) (accession number OL405009) clustered with *Polygonatum* species in their phylogenetic analysis and were labeled as *Polygonatum alternicirrhosum* Hand.-Mazz. However, data from other sources (NC_088740, OP428712, OP428713, OP428714, OP428717) support Floden (2014b) reclassifying it as *Heteropolygonatum alternicirrhosum* (Hand.-Mazz.) Floden. Therefore, we suspect that the sample corresponding to OL405009 may represent a misidentified sample.

#### Additional specimens examined (Paratypes).

China • Guizhou Province, Zunyi City, Xishui County, Xishui National Nature Reserve, 28°09'N, 105°54'E, 1200 m a.s.l., 22 July 2024, Xiao Wang Wx20250010 (KUN [1729523]!).

## Supplementary Material

XML Treatment for Heteropolygonatum xishuiense

## Additional information

### Use of AI

No use of AI was reported.

### Author ORCIDs

Xiao Wang https://orcid.org/0009-0006-1253-4095

Miao-Miao Wang https://orcid.org/0000-0002-2194-2959

Meng-Qi Han https://orcid.org/0000-0002-9059-7255

### Data availability

All of the data that support the findings of this study are available in the main text.
